# The Effect of Kinesiology Taping on Posture, Balance, and Gait in Patients Suffering from Low Back Pain

**DOI:** 10.3390/diagnostics14222506

**Published:** 2024-11-09

**Authors:** Józef Alphons Opara, Tomasz Fiałkowski

**Affiliations:** 1 Institute of Physiotherapy and Health Sciences, Jerzy Kukuczka Academy of Physical Education, 40-065 Katowice, Poland; 2 InterHealth Canada—Szpital Żywiec, 34-300 Żywiec, Poland; tomasz_f@poczta.onet.pl

**Keywords:** Kinesio taping, low back pain (LBP), body posture control, balance, gait, coordination

## Abstract

Background: Low back pain (LBP) is the leading cause of lost productivity, absenteeism, disability, and early retirement worldwide. LBP treatment should be comprehensive, including prevention, education, diagnosis, and various treatment methods. Management can be divided into pharmacological and non-pharmacological. The role of physiotherapy in the comprehensive treatment of LBP is very important. Elastic therapeutic tape, also called kinesiology tape or therapeutic kinesiology tape, has been used for about 50 years. Purpose: The aim of this study was to evaluate the effectiveness of Kinesio taping in patients suffering from (LBP), and its possible effect on the swing of the center of pressure (COP), balance, gait, improvement of coordination, and pain reduction. Methods: A total of 60 patients aged 20 to 83 years (54% women) were examined, all of whom fulfilled the requirements for admission and rejection. The L1–L5 spine of the experimental group underwent Kinesio taping in addition to thorough rehabilitation. The control group underwent balance control training based on visual feedback (VFB). The research tools used included the Bohannon single-leg standing test, the FAC (Functional Ambulatory Category) scale, the Podsiadlo and Richardson Standing and Walking Test called “Timed Up and Go” (TUG), evaluation of lumbar spine movement, Lasegue’s test and examination of neurological deficit symptoms, and self-assessment of pain using the Visual Analog Scale (VAS). Results: The effects of Kinesio taping on pain, gait, coordination, postural control, and balance are statistically significant. The main parameter influencing the effects of Kinesio taping was age (below 55 years); this relationship was also observed in the control group. Conclusions: Kinesio taping is an effective method in the treatment of LBP. It improves postural control, balance, gait, coordination, and pain.

## 1. Introduction

Low back pain (LBP) is commonly classified as a lifestyle disease. According to the World Health Organization (WHO), low back pain (LBP) affected more than 619 million people worldwide in 2020, and the number of cases is expected to increase to 843 million cases by 2050, mainly due to population expansion and aging [1]. LBP lasting more than 12 weeks is a chronic disease and is associated with the risk of sleep problems, depression and/or anxiety, and often disability. LBP is the leading cause of years lived with disability (YLD) and a serious challenge for primary health care worldwide [2]. The treatment of LBP is very complex and should be comprehensive, including prevention, education, diagnosis, and various treatment methods [3]. The role of physiotherapy in the comprehensive treatment of LBP is very important.

Alves et al. compared the clinical care standards for LBP in Australia, Canada, and the United Kingdom. These three standards provide consistent recommendations in quality statements regarding imaging, patient education/counseling, and self-management. However, the three clinical care standards differ in statements regarding psychological assessment, opioid analgesics, non-opioid analgesics, and non-pharmacological therapies [4].

LBP treatment can be divided into medication-based and non-pharmacological. It is impossible to imagine this treatment without physiotherapists. Jayani et al., based on a systematic review of the literature on physiotherapists’ attitudes and beliefs towards LBP, recently concluded that we still need to develop additional interventions to address attitudes and beliefs that will ultimately support the updating of the biopsychosocial model of care in LBP physiotherapy [5].

Born in 1940, Dr. Kenzo Kase created a tape and therapeutic taping technique in 1973 that could support muscle and joint function without reducing or limiting range of motion. When developing the Kinesio taping method (KT), he also took into account its impact on the lymphatic system. After two years of research on KT, applying Kinesio tapes to muscles, and examining their elasticity, adhesion, and breathability, Dr. Kase developed the Kinesio taping method (Kinesio^®^ Taping) and taping patches (Kinesio Tex Tape), which are still used today and help in many diseases [6].

Trobec and Peršolja (2019), based on a comprehensive review of the literature, stated that the effect of KT in reducing pain in LBP is beneficial but was not statistically significant in the analyzed studies. This therapy can be used as a complement to conventional physiotherapy procedures and may be important for patients due to its easy availability and safety [7]. Based on a systematic review and meta-analysis of nine RCTs, Li et al. (2023) concluded that KT may have an immediate and short-term beneficial effect on reducing pain intensity, but existing evidence does not support the superiority of KT over other interventions in improving function in people with chronic LBP [8]. In 2024, Elabd AM and Elabd OM described the efficacy of KT added to lumbar stabilization exercises (LSE) on adult patients with mechanical LBP. Fifty subjects have been included in a randomized, single-blind clinical trial. The authors concluded that the combination of KT and LSE is an effective treatment option for chronic mechanical LBP. Although patients in both study groups (LSE and LSE + KT) showed significant changes in all outcomes, the combined interventions induced more significant reductions in back disability and pain intensity [9].

These are few articles on visual feedback (VFB) in LBP. Meinke et al. published in 2022 the results of a randomized controlled trial that was conducted with the intervention group (*n*-14) receiving unsupervised home exercises with VFB using the Valedo Home, an exercise based on three inertial measurement units placed with medical adhesive strips at the height of the spinal process of the S1 and L1 vertebrae and on the left leg, 20 cm from the lateral femoral condyle. The control group (*n*-13) received no intervention. The intervention had no significant effect on postural balance or other outcomes (pain intensity, disability, quality of life, and fear of movement). Still, the wide range of adherence and a limited sample size challenged the robustness of these conclusions. The authors stated that future work should focus on improving adherence to digital interventions [10].

The aim of our study was to evaluate the effectiveness of KT in patients suffering from LBP. In our first article, we presented the effect of balance training with visual feedback (VFB) on posture and balance in patients suffering from sciatica [11]. Now, we present the effect of KT on center of pressure (COP) sway, balance, gait, coordination, and pain in patients suffering from LBP.

## 2. Methods

### 2.1. Patients

Sixty patients suffering from chronic LBP for 6–24 months (54% women) aged 20 to 83 years (mean 54) met the requirements for inclusion and exclusion (Table 1). In the KT group, the average age was 56.5 years, and in the VFB group, 55.2 years. The study was initiated after obtaining permission from the local Bioethics Committee (Komisja Bioetyczna przy Akademii Wychowania Fizycznego w Katowicach, Decision Number 5/16). The study was conducted in a sanatorium-hospital complex for rehabilitation purposes.

### 2.2. Inclusion and Exclusion Criteria

Inclusion criteria: adults suffering from LBP with disk–root conflict, according to the Bernard Jr. and Kirkaldy–Willis classification belonging to group A1 (herniation of the nucleus pulposus), patients belonging to the QTF 6 category according to the Quebec Task Force classification, i.e., spinal root compression confirmed by imaging techniques such as CT or MRI, patients meeting the QTF criteria: QTF 3—low back pain with radiation to the limb below the knee, QTF 4—low back pain with radiation to the limb with neurological symptoms, and QTF 9—condition after surgery in the lumbar-sacral section for more than 6 months [12,13].

Exclusion criteria from the study: not meeting the criterion of belonging to group A1 according to the Bernard Jr. and Kirkaldy–Willis classification, not meeting the QTF 6 criterion (no root compression), not meeting the QTF 3, QTF 4, and QTF 9 criteria (no radiation, no neurological symptoms, time less than 6 months after surgery), patients with abnormalities suggesting an inflammatory factor in laboratory tests, coexisting diseases of the cardiovascular, respiratory, connective tissue, hormonal, metabolic, extrapyramidal, psychosis, and addiction syndromes; significant deformations and defects of the musculoskeletal system (shortening of the lower limb by more than 3 cm, complete stiffening of the ankle, knee, or hip joint, significant scoliosis, etc.); previous knee and/or hip joint endoprosthesis. Twenty-four of the sixty patients had spine surgery.

### 2.3. Intervention

In the first group (KT), Kinesio taping was applied to the lumbar area (L1–L5) in addition to thorough stationary rehabilitation (about three hours per day) for three weeks (six days per week). Two thin, cotton, porous, elastic KT tapes, 5 cm wide, were applied to the trunk muscles in a standing position with 75% tension along the lumbar spine from the level of the L1 to L5 vertebrae, parallel on both sides of the spine, and maintained for four days. This procedure was performed three times during the stay. The break between individual applications was three days, during which the patient did not wear the tapes. The second group (VFB) received balance control training with visual feedback in addition to thorough rehabilitation. For this purpose, the “tracking” program on the ALFA platform was used.

In both groups, on the first and last day of their stay in the ward, a static and dynamic stabilometric test was performed using the ALFA platform. The static test consisted of assessing the so-called load distribution on the left and right limbs within 30 s. The patient stood on a special square plate with built-in pressure sensors. The device cooperated with a computer and was equipped with an LCD monitor. The result was given in percentages and helped assess the progress of the patients’ rehabilitation based on the above assumptions (the patient loads less on the side where the pain occurs). The dynamic test, which lasted 30 s, consisted of using training, which in both groups also assessed the progress of rehabilitation. The dynamic test was performed using a program called “tracking”. It was performed on the first and last day of their stay in the ward. In the second group—VFB—the “tracking” program was used for training. The subject stood on a stabilometric platform and observed a point on the monitor, the position of which reflected the projection of the center of gravity of his body on the base plane, scaled according to the way of loading the lower limbs applied during the exercise. The rehabilitated subject performed body movements as a result of which the point reflecting the scaled projection of the center of gravity of his body followed the stimulus drawn by the software. The distance covered by the center of gravity of the body on the screen in following the escaping virtual point was recorded graphically and digitally (in centimeters). If both points coincided during the test, this was recorded as the “time of target residence”. The result was presented as a percentage of the 30 s time. Static and dynamic (follow-up) stabilometry tests were conducted in the same room and at the same time. The conditions in the room were the same on both the first and last day of the study (temperature, light intensity, equipment setup). Each patient was tested in the same environment on both the first and last days. The VFB training lasted 10 min, was performed five times a week, and lasted three weeks.

### 2.4. Outcome Measures

The outcome was measured using the Bohannon one-leg stance test, the Functional Ambulatory Category (FAC) scale, the Podsiadło and Richardson Timed Up and Go (TUG) scale [14,15,16], the Schober lumbar spine mobility assessment, the Lasegue sign, the examination of neurological deficit symptoms, and self-assessment of pain using the Visual Analog Scale (VAS) [17].

### 2.5. Statistical Evaluation

For statistical evaluation, we used Pearson’s chi-squared test, the Kendall rank correlation coefficient, and the Wilcoxon rank-sum test.

## 3. Results

Table 2 displays the COP sway (measured in centimeters) and coincidence with the moving point (a virtual stimulus) over the course of the 30 s analysis (%).

Table 3 displays the distribution of limb loads and the amount of time spent standing on one leg with eyes open.

Table 4 displays the post-intervention changes in the lumbar spine mobility (Schober), Laségue’s sign, Functional Ambulatory Category (FAC), Timed Up and Go test (TUG), and VAS.

Table 5 presents the results in the KT group after applying the Mann–Whitney U test to the “Age” variable.

Table 6 presents the results in the VFB group after applying the Mann–Whitney U test to the “Age” variable.

### Summary of Results

The results are presented in Table 2, Table 3, Table 4, Table 5 and Table 6. Both dynamic KT and VFB had statistically significant effects on postural control, balance, gait, coordination, and pain. No effect of gender was observed. In both groups, the statistically significant effect of the intervention on balance was better in participants under 55 years of age. No side effects of either KT or VFB were observed.

## 4. Discussion

Despite the initial skepticism, the use of KT has continued to grow and is now widely used, especially in musculoskeletal disorders and injuries. In 2013, Kalron and Bar-Sela, based on a systematic review of the effect on musculoskeletal disorders, found moderate evidence for an immediate reduction in pain when wearing KT. However, there was no support for any long-term effect. Further research is clearly needed [18].

Here are some reports on the effects of KT on LBP. The first systematic review of KT for chronic LBP was published in 2016 by Nelson. She found five studies with 306 participants that met the inclusion criteria; the methodological quality of the included RCTs was good. Moderate evidence suggests that KT, either as a stand-alone treatment or in combination with another treatment, is not more effective than conventional physiotherapy and exercise in improving pain and disability outcomes. Limited evidence suggests that KT is more effective than sham taping in improving range of motion (ROM) and global perceived effect (GPE) in the short term. The author concluded that KT does not replace traditional physiotherapy or exercise but may be most effective when used as an adjunct therapy, perhaps by improving ROM, muscle endurance, and motor control [19].

Sheng et al. published a systematic review and meta-analysis of KT for the treatment of chronic nonspecific LBP in 2019. The quality of eight studies (totaling 530 participants aged 18–80 years, including 257 men and 273 women) that met the inclusion and exclusion criteria was moderate. Patients with chronic nonspecific LBP in the KT group achieved better pain relief and improved daily activities than those in the control group. The authors concluded that KT, alone or in combination with other general physical therapies, is superior to other general therapies or placebo patches in the treatment of chronic nonspecific LBP, providing greater pain relief and improved daily activities [20].

Another systematic review and meta-analysis were presented in the same year by Li et al. They found a total of 10 articles with 627 participants: 317 in the KT group and 310 in the control group. While KT was not superior to placebo patching in reducing pain, neither alone nor in combination with physiotherapy, it was able to significantly improve disability compared with placebo patching. Authors’ conclusion: because KT seems to be convenient to use, it may be used in people with chronic LBP in some cases, especially when patients could not obtain other physiotherapy [21]. Another systematic review and meta-analysis on the same topic presented also in 2019 by Luz Júnior et al. identified 11 RCTs (pooled *n* = 743). Very low to moderate quality evidence showed that KT was not superior to any other intervention for most outcomes assessed in patients with chronic non-specific LBP. The authors found no evidence to support the use of KT in clinical practice in patients with chronic, nonspecific low back pain. Level of evidence: 1 [22].

The first systematic review of the literature on COP deviation as a measure of balance performance in patients with nonspecific LBP compared with healthy controls was published in 2011 by Ruhe et al. Sixteen articles met the inclusion criteria. The majority of articles (14/16, 88%) found that patients with nonspecific LBP had increased mean COP velocity and overall deviation compared with healthy controls. This was statistically significant in most studies (11/14, 79%). Increased anteroposterior sway was also observed in patients with LBP. Patients with LBP exhibited greater postural instability than healthy controls, as evidenced by larger COP deviations and higher mean velocity. While reduced postural stability in LBP patients appears to be additionally related to the presence of pain, it does not seem to be related to the exact location and duration of pain. No correlation could be identified between pain intensity and the magnitude of COP deviations [23].

In a recent study published in 2024, Sung and Lee examined trunk stability and postural control in sway parameters in people with and without persistent low back pain. A total of 39 control volunteers and 26 LBP patients took part in the research. In three unilateral standing trials, the postural sway ranges, COP/COG (center of gravity) sway, and sway speeds (determined by dividing the route length by the time in the anteroposterior (AP) and mediolateral (ML) directions within 10 s) were examined. The difference in sway range following multiple trials showed significant group interactions. There were noteworthy group interactions in both directions during repeated trials with regard to the COG sway range. According to the authors’ findings, during the first two trials, the LBP group displayed lower ML sway speeds, which increased trunk stability. The COG findings demonstrated how trunk methods may be used to improve neuromuscular control and postural stability during unilateral stance [24].

In 2021, Jassi et al. reported the results of star-shaped KT (four intersecting tapes) compared with sham KT and minimal intervention (MI) on pain intensity and postural control in 120 individuals with chronic LBP aged 18–60 years. The primary outcome measures were pain intensity and mean COP sway velocity, and disability score (Oswestry Disability Index) was a secondary outcome. Results: Pain intensity was significantly lower in the star-shaped KT group than in the MI group immediately after the intervention and on the seventh day of the intervention. No significant differences were observed between groups in mean COP sway velocity and disability score throughout any of the intervals of follow-up. Author’s conclusions: There was no significant effect of the star-shaped KT intervention on pain intensity and postural control in individuals with LBP compared with MI or sham KT. The results of this study suggest that the benefits of KT are more likely to be attributed to contextual factors than to specific taping parameters [25]. The authors also wondered about the pain-relieving mechanism of KT—it may lift the skin of the joint or muscle of interest, thus allowing for better circulation and lymphatic drainage. It works by relaxing the tension of sensory receptors. Regarding differences in the mechanism of musculoskeletal disability, they cited the work of Bagheri et al. (2018), according to which KT may work by supporting and increasing the electrical activity of muscles. They included 20 physically inactive, healthy male participants aged 22–45 years. Only men were preferred because the excitability of motor neurons could be modulated by regular exercise and could be influenced during the luteal phase. The five experimental sessions included: control without KT or Eutectic Mixture of Local Anesthetics (EMLA); EMLA only; kinesiology tape only; sham tape only; and kinesiology tape and EMLA treatment. H-reflex parameters were facilitated by kinesiology tape with and without EMLA; however, EMLA inhibited H-reflex parameters in both the soleus and lateral gastrocnemius muscles. Sham tape did not change the H-reflex recruitment curve parameters. The statistical model showed a significant difference between KT and sham tape sessions and control sessions, between KT-EMLA and EMLA, and between KT-EMLA and the control session. Author’s conclusions: KT facilitates muscle activity, and the mechanism underlying the soleus motor neuron pool involves cutaneous receptors [26].

Recently, Tran et al. published a systematic review and meta-analysis of the effectiveness of KT compared with other treatments for musculoskeletal disorders. A total of 36 studies were included in the quantitative analysis. KT improved both pain and disability when applied to any body region. Within the first five days of treatment, KT significantly reduced pain in all body regions and also after four to six weeks of treatment. When applied to disability in patients with LBP, KT significantly reduced disability within five days of treatment. Furthermore, KT showed improvement in disability in all body regions after four to six weeks of treatment. According to the authors, these findings support KT as an adjuvant to other treatments for musculoskeletal disorders [27].

It is well known that individuals with LBP may have poorer motor control compared to their healthy counterparts. In 2019, Ge et al. published a systematic review and meta-analysis on the impact of LBP on balance in older adults. Thirteen case–control studies comparing balance parameters in individuals with LBP and healthy individuals were included. The experimental group (LBP) was found to be associated with a significantly larger COP area, faster COP sway velocity in the anteroposterior and mediolateral directions, longer anteroposterior path, slower walking speed, and longer TUG time than the control group. Conclusions: These results showed that balance was impaired in older adults with LBP [28].

People with LBP frequently have their trunk and lower limb biomechanics while walking and running evaluated. It can be expected that LBP affects both qualitative and quantitative gait parameters. Smith et al. published a systematic review and meta-analysis of walking in people with LBP in 2022. Ninety-seven studies were included. Compared with people with a normal spine, people with persistent LBP walked more slowly and had a shorter stride. Individuals with low back pain did not exhibit any variations in the amplitude of mobility of their hips, pelvis, or thoracic or lumbar spine. The biomechanics of running did not consistently differ among the groups. Conclusion by the author: There is weak to moderate evidence that the gait of those with chronic low back pain differs from that of persons with normal spines [29].

Pain is the primary cause of suffering for the patient. LBP encompasses a spectrum of different types of pain (e.g., nociceptive, neuropathic, and nociplastic or nonspecific pain), which often overlap. Various instruments have been developed to assess two key dimensions of pain experience—pain intensity (how much the person suffers) and pain impact (how much the person suffers). The most popular are: the Visual Analog Scale (VAS) [13], the Numerical Rating Scale (NRS), the McGill Pain Questionnaire (1972) modified to a short form (1987), the Aberdeen Low Back Pain Scale (1994), and the Von Korff Graded Chronic Pain Scale (1992) modified in 2000 [30,31,32,33,34,35].

The best measure of outcome in LBP treatment seems to be disability assessment. Recently, Chmielewski and Wilski described the psychometric properties of five of the most popular and well-known measures currently used to assess disability in LBP: the Oswestry Disability Index (ODI), the Roland–Morris Disability Questionnaire (RMDQ), the Quebec Back Pain Disability Scale (QBPDS), the Low Back Outcome Score (LBOS), and the Low Back Pain Rating Scale (LBPRS) [36,37]. The Roland–Morris Disability Questionnaire (RMDQ) can also be considered a quality of life questionnaire [38,39]. The primary method of comprehensive rehabilitation is exercise. Hayden et al. conducted a systematic review of the Cochrane database of exercise therapy for chronic LBP. They found 249 exercise treatment trials; 57% of the studies (142 trials) compared exercise therapy with a comparator that did not involve exercise, whereas 61% of the studies (151 trials) examined the efficacy of two or more distinct forms of exercise therapy. The mean age of the study participants was 43.7 years, and a mean of 59% of the study population was women. The majority of the trials were deemed to be biased, with 79% of them being at risk of performance bias due to challenges in blinding exercise treatment. Finally, the authors concluded that there was moderate-certainty evidence that exercise is probably effective for treating chronic low back pain compared with no treatment, usual care, or placebo for pain. The effect of exercise treatment compared with no treatment, usual care, or placebo comparisons was small for functional limitations, failing to meet our threshold for a minimally clinically important difference. In comparison to other conservative treatments, exercise also reduced pain (low-certainty evidence) and functional limitation ratings (moderate-certainty evidence); however, when all comparisons were taken into account at once, these benefits were negligible and not clinically significant. Exercise therapy was likely more successful than counseling, education, or electrotherapy alone, according to subgroup analyses, but there were no differences seen in the outcomes of manual therapy treatments [40].

Nwodo et al. conducted a review of reports of core stability exercises compared with conventional exercises for the treatment of chronic LBP. Core stability exercises (CSE) have gained popularity in recent years, but there is a lack of consensus on the best exercise treatment. A total of 14 RCTs were included in the analysis. The data indicated that core stability exercises were superior to conventional exercises for short-term pain relief. Ten studies included self-reports of specific back functional status, and compared with conventional exercises, core stability exercises resulted in significant improvements in function. The authors concluded that compared with conventional exercises, core stability exercises were more effective in reducing pain and improving physical function in people with CLBP in the short term, although only two studies conducted post-intervention follow-up assessments [41].

Saragiotto et al. enrolled 40 patients with chronic LBP in a randomized trial of stabilization exercises (SE) or a control group. Both groups received 12 sessions of routine physiotherapy for four weeks. The SE group also received intensive stabilization exercises. Balance (referring to the overall (OSI), anteroposterior (APSI), and medial–lateral stability (MLSI) indices) and functional disability were assessed using the Biodex Balance System with open and closed eyes. Both groups effectively demonstrated improved stability indices and functional abilities, as well as reduced pain intensity. The SE protocol made patients less dependent on vision, possibly due to improved stability. Since pain reduction did not differ between groups, the greater functional improvement in the SE group cannot be simply interpreted as pain interference and may be related to the patients’ postural control abilities [42].

Comparing our results to other investigations, one can also state similarities and contradictions. The main indication for using KT in LBP is pain. However, because LBP often involves disturbances in posture, balance, and gait, the indications for KT can be expanded, especially in chronic LBP. As was described above in the review of the literature, individual authors used different methods of application and outcome measures—primary and secondary. While in all cases KT had immediate and short-term positive effects on reducing pain intensity, functional effects, i.e., disability, balance, gait, posture, and trunk range of motion (ROM), were not so obvious. One can also observe a huge discrepancy in choosing the control group—only in one case was sham KT used. In our study, KT proved to be effective in postural control, balance, gait, coordination, and pain. We also observed a statistically significant better effect of both interventions (KT and VFB) on balance in participants under 55 years of age. It is also worth emphasizing that no side effects of either KT or VFB were observed. To summarize: further studies are needed, including RCTs with sophisticated protocols and scientifically designed multicenter studies, on large samples, to improve our knowledge of the application KT in LBP.

## 5. Conclusions

Both Kinesio taping and visual balance feedback control training have statistically significant effects on postural control, balance, gait, coordination, and pain in people suffering from LBP. The main parameter with a statistically significant effect in both interventions was age—subjects younger than 55 years achieved better results. No side effects were observed.

## Figures and Tables

**Table 1:** General characteristics of the subjects.

		KT Group		VFB Group	
		*n*	%	*n*	%
Gender	Females	16	27%	16	27%
	Men	14	23%	14	23%
Age (years)		20–83		36–80	
Mean age (mean ± SD)		53.9 ± 14.65		55.2 ± 13	
Body length (mean ± SD) cm		170.10 ± 19.59		169.17 ± 7.18	
Body mass (mean ± SD)		79.53		80.37 ± 15.42	
BMI (average)		27.49		28.08	

**Table 2:** COP sway and coincidence with moving point.

	KT Group			VFB Group		
Parameter Name	Pre Intervention	Post Intervention	*p* Value	Pre Intervention	Post Intervention	*p* Value
COP sway (centimeters) with eyes open	175.53	216.96	0.001	216.19	229.31	0.14
Execution time (%)	16.37	30.50	0.00002	18,93	36.93	0.00001

**Table 3:** Distribution of limb loads and duration of standing with eyes open on one leg.

	KT Group			VFB Group		
Parameter Name	Pre Intervention	Post Intervention	*p* Value	Pre Intervention	Post Intervention	*p* Value
Limb load distribution L/R leg (%)	L: 49.93 R: 50.07	L: 50 R: 50	0.69	L: 49.97 R: 50.03	L: 49.40 R: 50.60	0.21
Standing time (s) on left leg	8.48	12.92	0.000002	6.04	9.62	0.000002
Standing time (s) on right leg	8.53	13.16	0.000003	6.49	10.75	0.000002

**Table 4:** Post-intervention changes in the VAS, Timed Up and Go test (TUG), Functional Ambulatory Category (FAC), Laségue’s sign, and lumbar spine mobility (Schober).

	KT Group			VFB Group		
Parameter Name	Pre Intervention	Post Intervention	*p* Value	Pre Intervention	Post Intervention	*p* Value
Schober test (cm)	2.85	4.28	0.00001	3.17	4.25	0.00003
Laségue (L)	53.87°	69.20°	0.000003	52.03°	67.50°	0.000003
Laségue (P)	55.17°	68.60°	0.000002	55.37°	69.90°	0.000002
FAC	4.83	4.97	0.067	4.60	4.90	0.01
TUG sec.	13.40	10.28	0.000004	14.24	11.35	0.00003
mean VAS	5.40	3.10	0.00001	5.87	3.50	0.00001

**Table 5:** Mann–Whitney U test for the “Age” variable in the KT group.

	Average Age > 55	Average Age < 55	SD Age > 55	SD Age < 55	*p* Value
TUG 1 parameter (tested on first day) ^a^	15.73	10.74	6.19	3.15	0.007
TUG 2 parameter (tested on last day) ^a^	11.78	8.57	3.39	2.23	0.011
COP sway interaction time with virtual target (%)—the first day of therapy ^b^	10.69	22.86	9.73	11.79	0.004
COP sway interaction time with virtual target (%)—the last day of therapy ^b^	22.31	39.86	16.28	20.39	0.004
Weight domination (left leg—first day of therapy) ^c^	41.31	70.43	35.45	34.86	0.032
Weight domination (right leg—first day of therapy) ^c^	58.69	29.57	35.45	34.86	0.032
Bohannon test (left leg, eyes open, the first day of therapy) ^d^	102.44	102.64	0.51	0.50	0.002
Bohannon test (right leg, eyes open, the first day of therapy) ^d^	102.19	102.29	0.40	0.47	0.042
Bohannon test (left leg, eyes closed, the first day of therapy) ^d^	4.79	12.70	3.22	7.33	0.020
Bohannon test (right leg, eyes closed, the first day of therapy) ^d^	5.16	12.38	3.10	11.69	0.044
Bohannon test (left leg, eyes open, the last day of therapy) ^d^	2.56	4.23	2.52	2.40	0.005
Bohannon test (right leg, eyes open, the last day of therapy) ^d^	2.47	4.93	1.66	4.02	0.040
Bohannon test (left leg, eyes closed, the last day of therapy) ^d^	7.36	19.27	3.72	13.57	0.046
Bohannon test (right leg, eyes closed, the last day of therapy) ^d^	8.24	18.77	3.24	15.29	0.021

^a^ Timed Up and Go (TUG) test. ^b^ Center of Pressure (COP) sway test. ^c^ left/right leg weight load domination. ^d^ Bohannon test.

**Table 6:** Mann–Whitney U test for the “Age” variable in the VFB group.

Parameter	Average Age > 55	Average Age < 55	SD Age > 55	SD Age < 55	*p* Value
COP sway interaction time with virtual target (%)—the first day of therapy ^a^	13.87	24.00	10.22	12.60	0.040
COP sway interaction time with virtual target (%)—the last day of therapy ^b^	27.00	46.87	12.26	20.73	0.007
Bohannon test (left leg, eyes open, the first day of therapy) ^c^	102.27	102.20	0.59	0.41	0.026
Bohannon test (left leg, eyes closed, the last day of therapy) ^d^	7.74	11.50	5.06	6.55	0.014

^a^ COP sway interaction time with virtual target (%)—the first day of therapy. ^b^ COP sway interaction time with virtual target (%)—the last day of therapy. ^c^ Bohannon test (left leg, eyes open, the first day of therapy). ^d^ Bohannon test (left leg, eyes closed, the last day of therapy).

## Data Availability

The original contributions presented in the study are included in the article, further inquiries can be directed to the corresponding author.
